# Data in longitudinal randomised controlled trials in cancer pain: is there any loss of the information available in the data? Results of a systematic literature review and guideline for reporting

**DOI:** 10.1186/s12885-016-2818-8

**Published:** 2016-10-06

**Authors:** Odile Sauzet, Maren Kleine, John E. Williams

**Affiliations:** 1 AG3 Epidemiology and International Public Health, Bielefeld School of Public Health, Bielefeld University, Postfach 10 01 31, 33501 Bielefeld, Germany; 2 Department of Anaesthetics and Pain Management, Royal Marsden NHS Foundation Trust, London, UK

**Keywords:** Cancer pain, Longitudinal RCTs, Statistical analysis

## Abstract

**Background:**

Given the prevalence of untreated pain among cancer patients, there have been calls for more and better research in the domain. Increasingly, calls for less waste and more optimal use of trial data collected are being made. Waste of data includes non-optimal statistical analysis and non-presentation of interpretable effect size as a measure of effectiveness of an intervention which also enable comparisons across studies.

**Methods:**

We reviewed the recent literature on randomised trials on longitudinal cancer pain to identify sources of loss of data information by collecting material on the nature of outcomes collected, analysed, the method of analysis and what was presented as a result of the trial. Illustrated with real data, we propose some guidelines on how to adequately analyse longitudinal data and report the results using mixed models.

**Results:**

We identified some major source of data information loss, one of which is the transformation of a continuous pain outcome. Not adjusting for the collected outcome baseline value is moreover a source of bias. Multiple testing by analysing the data cross-sectionnally at each time-point leads to loss of information and power. Finally, effect sizes reflecting the effectiveness of the intervention were never reported.

**Conclusions:**

We identified several sources of information loss in the way longitudinal trials on pain were analysed and reported. However these problems could be easily solved by using regression methods like mixed models and presenting regression parameters to provide a concrete quantitative effect of the intervention.

**Electronic supplementary material:**

The online version of this article (doi:10.1186/s12885-016-2818-8) contains supplementary material, which is available to authorized users.

## Background

According to the ESMO Guideline working group [1] over 80 % of cancer patients with advanced metastatic disease suffer from pain. A vast literature [2–4] reports the inadequacy of pain treatment among these patients despite numerous initiatives and recommendations [5–8]. Therefore high quality trials assessing the efficacy of analgesic drugs and treatment strategies are required for this population of patients. Quality research includes optimally using all the data collected and analysing it an informative way i.e. using the statistical method which best reflects the effect of the intervention. Recently, it has been reported that there were numerous examples of waste in the running of clinical trials [9], non-optimal use of the data collected being one of them.

Repeated measures are often collected in cancer pain trials in order to reflect the need for lasting effects for the patients, the speed of action, or to evaluate a time to onset of relief. Longitudinal data allow comparisons of the dynamic of the intervention and the control. Statistical care needs to be taken because repeated measures on the same patient are not independent. But modern methods of analysis which permit to analyse such data like mixed model are now implemented in most statistical software packages which make them easily accessible to researchers. Advantages of mixed models include: i) the longitudinal nature of the data can be taken into account without loss of information as a multiple cross-sectional analysis would do ii) data for all patients with at least one measure post-baseline can be used for the analysis, and iii) the exact timing of the measure can be accounted for. Moreover the regression coefficient for group effect obtained can be used to present a quantitative value of the effect of the intervention in terms of pain measure.

Baseline pain measures need to be collected for several reasons. One is that patients with different levels of pain may be affected differently by the intervention. With this information missing, the real effect of the intervention might be over or underestimated [10]. Also because of the reduction to the mean (patients with higher pain score will see larger effects) baseline measures need to be controlled for in a regression model [11] and this despite an appropriate randomisation. While neither mentioning either baseline explicitly nor longitudinal studies, the IMMPACT [12] recommendations include reporting absolute and relative differences of pain measures from baseline.

The aim of this paper is two-fold. The first is to report the results of a systematic review on how longitudinal data in cancer pain in randomised controlled trials (RCT’s) is analysed. The aim is to see if there is any evidence of systematic loss of information due to suboptimal use of the data. Secondly, we provide guidelines on how to make the best use of the data collected and how to report results using regression parameters.

## Methods

In October 2013, the databases Medline, Medpilot, Cochrane Library, Scopus/SciVerse were searched for articles reporting RCT’s or protocols for RCT’s on the treatment of pain in cancer patients. RCT’s identified on pain produced by cancer diagnostic procedures and studies on postoperative pain were excluded from the review, as were systematic reviews. The languages were limited to English, French and German due to limited resources. Studies reporting a secondary analysis of RCT data were excluded as well as if an assessment of pain was made only as a part of a measurement of quality of life. In order to reflect recent practices we restricted our search to articles published in 2009 or later. The review was later updated to include articles until the year 2015. The MeSH terms are given in appendix. All extracted studies were screened for eligibility independently by two of the authors by reading the abstract. The full text of all eligible studies was obtained. The reporting of this review follows the PRISMA statement checklist [13].

Data were collected using a form piloted for consistency. Data were collected independently by two of the authors and when entries were in disagreement, the articles were further checked. The agreement considered good if any differences between reviewers could be resolved after checking the articles. The full list of items extracted from the studies can be seen in Tables 1, 2, 3. It included background information on the study, whether a baseline measure of pain was collected, whether the data was analysed longitudinally or cross-sectionally at each time-point and the method of statistical analysis. We also considered if the data was analysed as continuous or in a dichotomised form, and whether baseline measures were adjusted for.

**Table 1:** Description of studies

Pain outcome	Primary only	46 % (34/74)
	Secondary only	22 % (16/74)
	Primary and secondary	23 % (17/74)
	Unclear	9 % (7/74)
Primary pain outcome collected	NRS^a^	23 % (17/74)
	VAS^b^	28 % (21/74)
	Brief Pain Inventory	31 % (23/74)
	Other instruments	18 % (13/74)
Number of groups	Two	87 % (64/74)
Number of patients	Median (range)	80 (9–2046)
Type of study	Parallel design	43 % (32/74)
	Comparison placebo/usual care	57 % (42/74)
	Cross-over design	18 % (13/74)
Duration of follow-up	Under24h	16 % (12/74)
	Patient dependent	7 % (5/74)
	Other: median (range) of duration	7 (1–260) weeks
Type of pain episodes	Breakthrough	5 % (4/74)
	Background	95 % (70/74)

^a^ Numerical rating scale

^b^ Visual analogue scale

**Table 2:** Method of analysis

Primary pain outcome analysed	NRS^a^ or VAS^b^ measures	60 % (44/74)
	Change from baseline	24 % (18/74)
	Aggregated NRS or VAS	10 % (7/74)
	Dichotomised outcome	23 % (17/74)
Analysis of repeated measures	Cross sectional at each time-point	37 % (27/74)
	longitudinal	38 % (28/74)
Method of analysis		
Cross sectional	*t*-test	44 % (12/27)
	ANOVA/ANCOVA	19 % (5/27)
	Non parametric test	36 % (10/27)
*Correction for multiple testing due to repeated measures*		15 % (4/27)
Longitudinal	Mixed Model	25 % (7/28)
	Repeated measure ANOVA	43 % (12/28)
	GEE	1 % (4/28)
	Area under the curve	7 % (2/28)
	Unknown/other	10 % (3/28)
Results presented		
	Mean (SD) at each time-point per group	44 % (31/71^c^)
	F values only (for ANOVA/ANCOVA)	29 % (5/17)
	Proportions	59 % (5/17)
	Hazard ratio, odds ratio (with CI)	23 % (4/17)
	None	17 % (12/71^#^)

Only one study using longitudinal regression type analysis presented a parameter estimate for group effect

^a^ Numerical rating scale

^b^ Visual analogue scale

^c^ From 74 studies, three were protocols

**Table 3:** Use of baseline data in the analysis of pain outcome

Baseline data	Collected	91 % (67/74)
Description of the inclusion	Mentioned in Methods section	40 % (27/67)
	Baseline measure included	60 % (40/67)
	Not included	21 % (14/67)
	Unknown	19 % (13/67)
Method of inclusion	Difference from baseline (cross sectional)	76 % (13/17)
	Covariate (Cross sec. and long.)	29 % (8/28)
	Time-point (Long.)	30 % (7/23)
	Used to compute a dichotomised outcome	41 % (7/17)

This work is a systematic review of the literature and contains no research on humans; therefore no ethical approval is required. Results of the review are presented in descriptive tables with absolute and relative numbers of articles for each item. The discussion is illustrated with data from the Treat and Screen study [14], a RCT to evaluate the effectiveness of pain treatment protocol and screening for patients with head and neck cancer. All analysis were performed using Stata 12 (StataCorp. 2011).

## Results

**Fig. 1:**
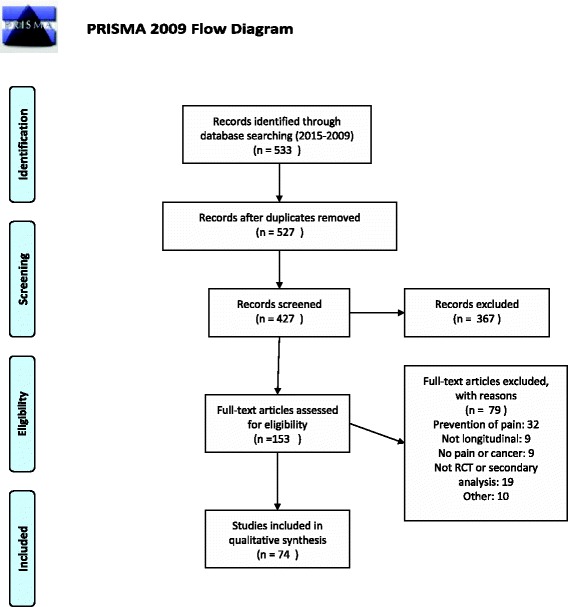
PRISMA flow diagram

We identified 74 eligible studies, three of which were protocols. Agreement between the two reviewers was good. The complete flowchart is given in Fig. 1 and a table summarising the data collected for each article in provided as Additional file 1. The study characteristics are presented in Table 1. Most studies identified concerned background pain (70/74) and only four focused on breakthrough pain. More than two thirds (69 %, 41/74) collected a pain measure as a primary outcome measure. The Brief Pain Inventory was the most used instrument (23/74, 31 %) followed by a visual analogue scale (VAS) (21/74, 28 %) and numerical rating scale (17/74, 23 %). However the continuous pain outcome is analysed as such in only 60 % (44/74) of studies, other using a dichotomised version (17/74), a difference from baseline (18/74) or an aggregated value (7/74). All results regarding the statistical analysis are presented in Table 2. Only 38 % (28/74) of studies performed a longitudinal analysis of the data. Other studies analysed the data cross-sectionnally (27/74), mostly at each measure time-point, thus losing the longitudinal information included in the data. Moreover repeated cross sectional analysis constitutes multiple testing for which only four studies reported using a correction. In the remainder, aggregated data or only one measure time-point was analysed thus losing completely the longitudinal nature of the study.

The data presented (for the 71 studies which were not protocols) included mostly mean and standard deviations at each time-point and for each group but in only one longitudinally analysed study were quantitative effects of the intervention presented as results of the trial.

Baseline data was collected in most, 91 % (67/74), but not all studies. In only 40 % (27/67) of studies, the method of adjustment for baseline data was reported in the Methods section. Moreover in 40 % of studies, it is either not known or it is clear that the baseline data was not adjusted for.

## Discussion

While some studies used an appropriate method for the analyse of longitudinal pain data, the present review revealed several sources of loss of information in longitudinal RCT’s on cancer pain. This means that there is a non-optimal use of the data collected is made. Thus more accurate information on the effect of an intervention is available but not known. If the choice of method of analysis does not necessarily affect the success of a trial if the latest is based on the significance of a statistical test, it affects the effect size and standard deviation presented. In meta-analysis, the cumulated loss of information could potentially make a difference in the recommendation made. This issue could be further researched but is not within the scope of this paper. After reviewing the list of highlighted problems–see also Table 4 for a summary–we show how they can be easily solved and how a researcher can present the output of interest without compromising on statistical optimality.

**Table 4:** Source, nature and solution to encountered loss of information

Source	Nature of the loss	Solution
Baseline data not adjusted for	Collected data not used, bias [9, 10]	Adjust for baseline in a regression model
Dichotomisation of the main continuous outcome	Loss of power, information [15, 16]	Analyse the continuous outcome as primary analysis, dichotomised outcome presented as secondary
Aggregated longitudinal data	Loss of the longitudinal nature of the data, loss of the treatment dynamic over time	Use a linear mixed model possibly with time as a covariate.
Cross sectional analysis at each time-point	Multiple testing requiring a correction, therefore loss of power	Use a linear mixed model with categorised time with time-group interactions.
No effect size provided	No information on the magnitude of the effect in term of outcome measure	Use a regression model (e.g. mixed model) and present the regression parameter for group effect with confidence interval

Information loss occurs when the continuous outcome collected is transformed before being analysed. Aggregated data is such that all the measures taken at different time-points on one patient are summarized to one value. This way the longitudinal nature of the data is lost, and either patients with missing data are left out or the aggregated values include unequal time-points leading to the outcome having varying meaning between patients. Dichotomisation is usually done when the primary outcome is the proportion of responders. Dichotomisation is a problematic practice because among other issues, it leads to a loss of power [15, 16]. This means that the number of patients to include in the study is much larger than if the continuous outcome was used in the primary analysis. Responder analysis or time-to-onset in pain studies should only be performed as a secondary analysis.

In half of the studies reviewed baseline outcome values were either not collected or not included in the analysis. As mentioned earlier adjusting for baseline data was necessary to control for the reduction to the mean and to obtain unbiased estimate of the effect of the intervention if it were to affect patients with different level of pain differently.

Analysing the data cross-sectionnally raises several issues. The first one is that the longitudinal nature of the data can only be accounted for heuristically by comparing the differences obtained at various time-points. Statistically, this involves multiple testing which needs to be corrected for therefore reducing the power. Also information on individual trajectories is lost. Some studies reported analysing difference from baseline in a longitudinal model. There are some conceptual difficulties in doing so because a difference in pain intensity between Week 1 and baseline and between Week 2 cannot necessarily be considered the same outcome. Instead the baseline outcome value should be adjusted for in the model. This review has made it clear that the method of analysis was not always the one which was making the best use of all the data available mostly by ignoring its longitudinal nature but also by using a method of analysis which leaves out any patient with missing values in the outcome as does repeated measure ANOVA.

We show how to analyse longitudinal data using linear mixed models but other regression methods exist [17]. These have the advantage of using all the data available from all patients who have at least one measure taken post baseline. Results of the trials should be presented as an effect size (measure of the effectiveness of the intervention) in terms of the regression parameter for the group effect and its standard error or confidence interval. We discuss three approaches which can be used to answer typical research questions in the field of chronic pain research.

A Mean Model can be used to compare overall differences in pain score post-intervention between the groups. We have applied a mixed model on the mean pain severity of the BPI questionnaire from the Screen and Treat study data using time (continuous) as a covariate (optional) and adjusting for baseline outcome values to correct effect estimates for the reduction to the mean:

- *Mean model*: the mean difference in Mean Pain Severity between usual care and intervention adjusted for baseline values is 0.55 score points (confidence interval: [−0.05, 1.14]).

If no adjustment was made for baseline values, the effect would be of 0.43, a 20 % smaller effect than with the adjustment for baseline. Moreover, the estimate is less precise with a larger standard error (0.35 against 0.30) leading to a wider confidence interval. Such differences are to be seen in the presence of inhomogeneous patient groups, i.e. patients with high and patients with low pain scores at baseline [11, 18].

The Slope Model is suitable when the evolution of pain is of interest. This model provides an overall rate of change from baseline. It consists in comparing the slope of pain scores over time (continuous), starting at baseline. This is typically the situation in the treatment of breakthrough pain when the treatment starts at a maximum of pain and where the treatment with the fastest response is the best. The difference in slope between the groups is obtained by estimating time-group interactions with time being a continuous variable. In this case, baseline is an outcome time-point and does not need further adjustment.

- *Slope model:* the mean pain severity decreased by 0.060 score points per week more in the intervention group then in the usual care group (confidence interval [0.003, 0.117]).

Time can also be used as a categorical variable with group-time interactions to obtain a separate group comparison at each time-point with adjustment for outcome baseline values. This is a more accurate and powerful alternative to multiple testing procedures in order to assess at which time-point the difference between groups is at its highest. This should be done as a secondary analysis after providing an overall mean difference over time (mean model above).

- *Categorical time variable:* The difference in mean pain severity at 1 month between usual care and intervention was 0.42 [−0.36, 1.20], at 2 months the difference was −0.25 [−0.90, 0.39] less than at 1 month and at 3 months 0.29 [−0.36, 0.95] more than at 1 month.

### Limitations

This review focused on the primary statistical analysis and not on the adequacy of the pain measure or the results obtained. It is clear that many studies did not use a validated instrument for chronic pain (only a third used the Brief Pain Inventory with the vast majority of studies using VAS od NRS in isolation ignoring the history of pain [10]) while most longitudinal studies analysed background pain which is a form of chronic pain. This point would require further work because of the bias incurred from the inaccuracy of pain measures but goes beyond the purpose of this work. This study did not show any indication that there was a relationship between the choice of pain measure and the method of analysis. However further research could be perform to show if there are any relationship between the pain measure and the effects shown by the study.

## Conclusions

Our review highlighted that the way the data was often analysed or the results presented in the clinical trials literature on cancer pain led to loss of some of the information present in the data collected. In order to present the best evidence available on treatments these practices should be avoided. Without compromising on the impact and interest that research studies generate, we have provided some indications on how methodology could be improved. In particular we have demonstrated how to avoid dichotomisation or multiple testing in the primary analysis and how to present informative effect as the result of the trial.

## Additional file

Additional file 1: Data_final_supp_material: this file contains a table with a summary of all the data collected (data collection form) for each article included in this review. (PDF 42 kb)

## Abbreviations

BIP: Brief Pain Inventory
NRS: Numerical analogue scale
VAS: Visual analogue scale

## References

[1] Jost L, Roila F (2010). Management of cancer pain: ESMO Clinical Practice Guidelines. Ann Oncol Off J Eur Soc Medl Oncol/ESMO.

[2] Deandrea S, Montanari M, Moja L, Apolone G (2008). Prevalence of undertreatment in cancer pain. A review of published literature. Ann Oncol Off J Eur Soc Med Oncol/ESMO.

[3] Fisch MJ, Lee J, Weiss M (2012). Prospective, observational study of pain and analgesic prescribing in medical oncology outpatients with breast, colorectal, lung, or prostate cancer. J Clin Oncol Off J Am Soc Clin Oncol.

[4] van den Beuken-van Everdingen MHJ, de Rijke JM, Kessels AG (2007). Prevalence of pain in patients with cancer: a systematic review of the past 40 years. Ann Oncol Off J Eur Soc Med Oncology/ESMO.

[5] IASP/EFIC. Unrelieved pain is a major global healthcare problem. http://www.iasp-pain.org/files/Content/ContentFolders/GlobalYearAgainstPain2/20042005RighttoPainRelief/factsheet.pdf. Accessed 03 Oct 2016.

[6] Gordon DB, Dahl JL, Miaskowski C (2005). American pain society recommendations for improving the quality of acute and cancer pain management: American Pain Society Quality of Care Task Force. Arch Intern Med.

[7] Ripamonti CI, Santini D, Maranzano E (2012). Management of cancer pain: ESMO Clinical Practice Guidelines. Ann Oncol Off J Eur Soc Med Oncology/ESMO.

[8] The British Pain Society. Cancer Pain Management. A perspective from the British Pain Society, supported by the Association for Palliative Medicine and the Royal College of General Practitioners 2010. http://www.britishpainsociety.org/book_cancer_pain.pdf. Accessed 03 Oct 2016.

[9] Ioannidis JPA (2014). Clinical trials: what a waste. BMJ.

[10] Breivik H, Borchgrevink PC, Allen SM (2008). Assessment of pain. Br J Anaesth.

[11] Vickers AJ, Altman DG (2001). Analysing controlled trials with baseline and follow up measurements. BMJ.

[12] Dworkin RH, Turk DC, Farrar JT (2005). Core outcome measures for chronic pain clinical trials: IMMPACT recommendations. Pain.

[13] Moher D, Liberati A, Tetzlaff J (2009). Preferred Reporting Items for Systematic Reviews and Meta-Analyses: The PRISMA Statement. Ann Intern Med.

[14] Williams J, Peacock JL, Gubbay A (2015). Routine screening for pain combined with a pain treatment protocol in patients with cancer: a randomized controlled trial. Br J Anaesth.

[15] Altman DG, Royston P (2006). The cost of dichotomising continuous variables. BMJ.

[16] Ragland DR (1992). Dichotomizing continuous outcome variables: dependence of the magnitude of association and statistical power on the cutpoint. Epidemiology.

[17] Twisk JWR. Applied longitudinal data analysis for epidemiology: A practical guide. Cambridge: Cambridge Medicine; 2013.

[18] Sauzet O, Kleine M, Menzel-Begemann A (2015). Longitudinal randomised controlled trials in rehabilitation post-stroke: a systematic review on the quality of reporting and use of baseline outcome values. BMC Neurol.

