# Supplementary material for: Data in longitudinal randomised controlled trials in cancer pain: is there any loss of the information available in the data? Results of a systematic literature review and guideline for reporting
Source: BMC Cancer. 2016 Oct 6;16:771. doi: 10.1186/s12885-016-2818-8 (PMC5054541; doi:10.1186/s12885-016-2818-8)
Supplement: Additional file 1: — Data_final_supp_material: this file contains a table with a summary of all the data collected (data collection form) for each article included in this review. (PDF 42 kb) [file 12885_2016_2818_MOESM1_ESM.pdf]

2	Baczyk	2013	unclear	VAS	2	177
3	Cleeland	2013	primary	BPI	2	2046
4	Hayes	2013	primary	Neuropatic pa	3	194
7	Lauretti	2013	secondary	VAS	6	72
8	Mercadante	2013	unclear	BPI	2	70
10	Wang	2013	primary	VAS	2	40
11	Wong	2013	primary	Wong-Baker f	2	9
12	Yang	2013	unclear	VAS	2	100
14	Ahmedzai	2012	primary	BPI	2	184
15	Batstone	2012	secondary	Likert-scale	2	44
16	Gautam	2012	primary	VAS	2	239
19	Hardy	2010	primary & secondary	unknown	2	185
25	Logothetis	2012	unclear	BPI	2	1196
26	Lopez Sendin	2012	primary	BPI	2	24
28	Mishra	2012	primary	VAS	4	120
31	Moksnes	2012	secondary	BPI	2	42
32	Portenoy	2012	primary	BPI	4	360
33	Rauck	2012	primary	VAS	2	98
34	Sima	2012	primary	NRS	2	246
38	Yang	2012	secondary	VAS	2	76
39	Yeo	2012	secondary	VAS	2	102
41	Caffo	2011	secondary	BPI	2	95
42	Carvalho	2011	secondary	VAS	2	70
43	Chou	2011	secondary	BPI	2	68
45	Fallon	2011	primary & secondary	NRS	2	84
47	Kravitz	2011	primary	medical outco	2	307
48	LeBlanc	2011	primary	NRS	2	54
50	Moknes	2011	primary	NRS	2	42
51	Oldenmenger	2011	primary	BPI	2	73
52	Oudot	2011	primary	VAS	2	35
55	te Bouveldt	2013	primary & secondary	BPI	2	210
56	Wyse	2011	primary & secondary	Lickert-scale	2	98
62	Atahan	2010	unclear	VAS	2	100
63	Benett	2010	primary	NRS	2	24
68	Homsi	2010	primary	4 point scale	2	32
69	Israel	2010	primary	NRS	2	31
70	Jahn	2010	secondary	unknown	2	240
73	Kroenke	2010	primary	BPI	2	405
74	Leppert	2010	primary	VAS	2	40
75	Lovell	2010	secondary	BPI	4	217
76	Lyon	2010	unclear	BPI	3	35
77	Meuse	2010	primary	unknown	2	274
78	Mercadante	2010	primary	unknown	2	60
79	Nguyen	2010	primary	NRS	2	40
82	Portenoy	2010	primary	NRS	2	93
83	Ridgway	2010	secondary	NRS	2	41
86	Smith	2010	secondary	BPI	2	89
88	Vayne Bosser	2010	primary	VAS	2	18
92	Buttler	2009	primary	pain rating sca	2	124
94	Cheville	2009	primary & secondary	NRS	2	28
100	Johnson	2009	primary & secondary	BPI	3	65
101	Liu	2014	unclear	VAS	2	26
102	Macpherson	2015	secondary	BPI	2	34
103	Yousef	2014	primary	BPI	2	120
104	li	2014	primary & secondary	BPI	3	84
105	Thapa	2015	primary	VAS	2	32

106	Chen	2013	primary	NRS	2	60
107	Rief	2014	primary and secondary	NA	2	60
108	Salas	2014	primary & secondary	other	2	200
109	William	2015	primary & secondary	BPI	2	156
110	Zeng	2014	primary	VAS	2	60
111	chan	2014	secondary	BPI	2	174
112	Hadjieva	2014	primary & secondary	NRS	2	38
113	Imanaka	2014	primary and secondary	NA	2	100
114	Leenstra	2014	primary	other	2	155
115	Kress	2014	primary & secondary	NRS	3	496
116	Nishihara	2013	primary & secondary	NRS	3	37
117	Kosugi	2014	primary & secondary	NRS (diary)	2	136
118	Huang	2014	secondary	BPI	2	52
119	amr	2014	primary	VAS	2	119
120	Agarwal	2015	primary	VAS (dairy)	2	44
121	Khedr	2014	primary & secondary	VAS	2	34
122	Hershman	2015	primary & secondary	BPI	2	243
123	Wang	2014	secondary	NRS	2	40

parallel	no	0	no	other
parallel	no	75	no	other
placebo/usualcare	no	52	no	longitudinally
placebo/usualcare	no	3	no	longitudinally
parallel	no	4	no	cross sectionally at each tin
placebo/usualcare	no	2	no	longitudinally
placebo/usualcare	no	0	no	other
placebo/usualcare	no	52	no	cross sectionally at each tin
parallel	no	4	no	cross sectionally at each tin
placebo/usualcare	yes	52	no	cross sectionally at each tin
placebo/usualcare	no	7	no	longitudinally
placebo/usualcare	no	1	no	longitudinally
parallel	no	97	no	other
placebo/usualcare	no	2	no	longitudinally
placebo/usualcare	no	4	no	longitudinally
parallel	no	2	no	cross sectionally at each tin
placebo/usualcare	no	5	no	other
placebo/usualcare	yes	0	yes	other
placebo/usualcare	no	1	no	cross sectionally at each tin
parallel	no	260	no	cross sectionally at each tin
placebo/usualcare	no	26	no	cross sectionally at each tin
parallel	no	0	no	cross sectionally at each tin
parallel	no	4	no	longitudinally
placebo/usualcare	no	4	no	other
parallel	yes	0	yes	cross sectionally at each tin
placebo/usualcare	no	12	no	longitudinally
parallel	no	14	no	other
parallel	no	2	no	cross sectionally at each tin
placebo/usualcare	no	9	no	longitudinally
parallel	no	1	no	other
placebo/usualcare	no	52	no	longitudinally
parallel	no	13	no	longitudinally
parallel	no	26	no	other
placebo/usualcare	yes	0	no	cross sectionally at each tin
parallel	no	1	no	cross sectionally at each tin
placebo/usualcare	yes	1	no	other
placebo/usualcare	no	4	no	longitudinally
placebo/usualcare	no	52	no	longitudinally
parallel	yes	2	no	cross sectionally at each tin
placebo/usualcare	no	4	no	longitudinally
placebo/usualcare	no	6	no	longitudinally
parallel	no	12	no	other
parallel	no	8	no	longitudinally
placebo/usualcare	no	0	no	cross sectionally at each tin
placebo/usualcare	yes	0	yes	cross sectionally at each tin
parallel	yes	2	no	other
placebo/usualcare	no	12	no	longitudinally
placebo/usualcare	yes	1	no	longitudinally
parallel	no	52	no	longitudinally
placebo/usualcare	yes	8	no	cross sectionally at each tin
parallel	no	8	no	cross sectionally at each tin
placebo/usualcare	yes	2	no	longitudinally
placebo/usualcare	no	8	no	other
placebo/usualcare	no	4	no	cross sectionally at each tin
parallel	no	26	no	cross sectionally at each tin
parallel	no	13	no	cross sectionally at each tin

placebo/usualcare	no	1	no	cross sectionally at each tin
placebo/usualcare	no	13	no	longitudinally
placebo/usualcare	no	2	no	longitudinally
placebo/usualcare	no	13	no	longitudinally
parallel	no	0	no	other
parallel	no		no	cross sectionally at each tin
parallel	no	0	no	cross sectionally at each tin
parallel	no	8	no	other
placebo/usualcare	yes	0	no	longitudinally
placebo/usualcare	no	4	no	other
parallel	no	2	no	other
placebo/usualcare	yes	0	yes	cross sectionally at each tin
parallel	no	13	no	cross sectionally at each tin
parallel	no	65	no	cross sectionally at each tin
parallel	no	16	no	longitudinally
placebo/usualcare	no	4	no	longitudinally
placebo/usualcare	no	24	no	other
placebo/usualcare	no	1	no	longitudinally

	no	prop	yes	dif from base
dichotomised	N/A	HR-CI	yes	dicho
GEE	N/A	m-CI	yes	time point
repeated measure ANOVA	N/A	nothing	no	
other	no	msd	yes	dif from base
repeated measure ANOVA	no	msd-F-values	yes	
aggregated for all measures Pre/Post	no	msd	yes	
t-Test	no	msd	yes	
NA	no	mdiff-CI	yes	cov
t-Test	no	msd	no	
other	N/A	F-values	no	
mixed model	no	msd-CI	yes	
dichotomised, time to event	no	HR-CI	yes	dicho
repeated measure ANOVA	yes	F-values	yes	time point
repeated measure ANOVA	yes	F-values	yes	
other	no	msd-CI	yes	
dichotomised	no	or	yes	dicho
aggregated	yes	msd	yes	cov
ANOVA	no	msd	yes	dif from base
t-Test	no	msd	yes	
t-Test	no	nothing	yes	
other	unclear/ unkn	chgbas	yes	dif from base
AUC	N/A	nothing	no	
not analysed as outcome	N/A	coef-se	yes	
ANOVA	no	msd	yes	dif from base
mixed model	N/A	coef-se	yes	cov
aggregated + dichotomised	unclear/ unkn	logrank	yes	dicho
other	unclear/ unkn	mdiff-CI	yes	
other	N/A	mean	yes	dif from base
aggregated	no	msd	no	
repeated measure ANOVA	N/A	Protocol	yes	
GEE	no	chgbas	yes	dicho
time to event	no	prop	yes	
other	no	msd	yes	dif from base
other	no	nothing	yes	
aggregated	N/A	F-values	no	
mixed model	no	Protocol	yes	
mixed model	N/A	nothing	yes	cov
t-Test	no	msd	yes	
GEE	N/A	msd	yes	cov
mixed model	N/A	miqr	yes	
used to calculate responses	N/A	prop	yes	dicho
repeated measure ANOVA	N/A	nothing	yes	
other	no	cohen	yes	
ANOVA	no	msd	yes	dif from base
aggregated	yes	msd-CI	yes	cov
mixed model	N/A	t-values	yes	
repeated measure ANOVA	N/A	msd	yes	
other	no	m-prop	yes	time point
other	N/A	mean	yes	dif from base
other	no	msd	yes	dif from base
repeated measure ANOVA	N/A	msd	yes	N/A
aggregated	N/A	nothing	yes	diff from base
t-Test	no	msd	yes	dif from base
t-Test	no	nothing	yes	N/A
t-Test	no	propt	yes	diff from base

t-Test	yes	msd	yes	diff from base
repeated measure ANOVA	NA	msd	yes	NA
repeated measure ANOVA	no	Protocol	yes	time point
mixed model	yes	msd	yes	cov
chi-squared-test	no	propt	yes	N/A
t-Test	no	nothing	unclear	
ANOVA	no	msd	yes	diff from base
	NA	propt	yes	dicho
AUC	yes	nothing	yes	time point
odds ratio	N/A	OR	yes	N/A
non-parametric test	no	msd	yes	diff from base
t-Test	no	msd	yes	N/A
ANOVA	no	msd	yes	N/A
t-Test	no	msd	yes	dif from base
repeated measure ANOVA	NA	msd	yes	time point
repeated measure ANOVA	N/A	msd	yes	cov
t-Test	N/A	none	yes	diff from base
GEE	NA	nothing	yes	time point

yes
yes
yes
no
yes
unclear/ unknown
unclear/ unknown
no
yes
N/A
N/A
unclear/ unknown
yes
yes
unclear/ unknown
no
yes
yes
yes
no
unclear/ unknown
yes
unclear/ unknown
NA
yes
yes
yes
no
yes
no
unclear/ unknown
yes
no
yes
no
N/A
unclear/ unknown
yes
no
yes
unclear/ unknown
yes
unclear/ unknown
no
yes
yes
unclear/ unknown
no
yes
yes
yes
unclear/ unknown
yes
yes
no
yes

yes
NA
yes
yes
no
unclear/ unknown
yes
yes
yes
no
yes
no
no
yes
yes
yes
yes
yes
